# Globalization's effects on world agricultural trade, 1960–2050

**DOI:** 10.1098/rstb.2010.0131

**Published:** 2010-09-27

**Authors:** Kym Anderson

**Affiliations:** School of Economics, University of Adelaide, Australia

**Keywords:** globalization, trade costs, distorted incentives, agricultural protectionism, trade policy reforms

## Abstract

Recent globalization has been characterized by a decline in the costs of cross-border trade in farm and other products. It has been driven primarily by the information and communication technology revolution and—in the case of farm products—by reductions in governmental distortions to agricultural production, consumption and trade. Both have boosted economic growth and reduced poverty globally, especially in Asia. The first but maybe not the second of these drivers will continue in coming decades. World food prices will depend also on whether (and if so by how much) farm productivity growth continues to outpace demand growth and to what extent diets in emerging economies move towards livestock and horticultural products at the expense of staples. Demand in turn will be driven not only by population and income growth, but also by crude oil prices if they remain at current historically high levels, since that will affect biofuel demand. Climate change mitigation policies and adaptation, water market developments and market access standards particularly for transgenic foods will add to future production, price and trade uncertainties.

## 1. The issue

One of the most striking features of economic development is the relative decline in the agricultural sector in growing economies. Also typical for countries with above-average population density is a decline in their agricultural comparative advantage as capital accumulation and industrialization proceed. An export-led boom in another sector, or large prolonged inflows of foreign aid, also weaken the international competitiveness of a country's farm sector. Changes in consumption patterns (the slow growth in consumption of farm products and, in middle-income countries, the move away from grains and other staples and towards livestock and horticultural products) also alter the net trade situation of countries. However, whether that leads to a decline or a rise in the overall food self-sufficiency in and net exports of total agricultural products depends also on productivity growth in farming relative to non-agricultural production (Anderson 1987), and in trends in government assistance to farmers relative to producers of other tradables. In the past, price-distorting policies have gradually changed from disfavouring to favouring agriculture relative to other tradable sectors as *per capita* incomes grow (Anderson 2009); globally, productivity growth has been faster in the farm sector than in other sectors (Martin & Mitra 2001).

A further influence on agricultural trade has been the acceleration of globalization over the past quarter-century. That has been characterized by a rapid decline in the costs of cross-border trade in farm and other products, driven by declines in the costs of transporting bulky and perishable products long distances, the information and communication technology (ICT) revolution and major reductions in governmental distortions to agricultural trade. Together, these developments have boosted economic growth and reduced extreme poverty globally, and in the process altered global agricultural production, consumption and hence trade patterns.

This paper first examines the key drivers of the above developments over the past four or five decades and then draws on that analysis and recent events to suggest likely drivers of—and uncertainties associated with—global food and other agricultural trade trends over the next four decades.

## 2. Key drivers of change since 1960

The first part of this section summarizes the structural changes in global agricultural markets and trade since the 1960s. The second part outlines one set of drivers, namely rapid technological changes including those that have lowered trade costs for farm products over the past quarter-century. The third part summarizes reforms to agricultural and trade policies since the 1980s and economy-wide modelling results that suggest those reforms have more than halved the global trade- and welfare-reducing effects of price-distorting policies.

### (a) Structural changes in global agricultural markets

One of the most striking features of economic development is the relative decline of the agricultural sector in growing economies. Also typical for countries with a reasonably high population density is a decline in their agricultural comparative advantage as industrialization proceeds (or when another sector such as mining, manufacturing or services enjoys an export-led boom or there is a sustained inflow of foreign aid). There is a wide dispersion across regions of the world in the importance of agriculture in national GDP and employment, in endowments of arable land and fresh water as well as capital per worker, in the availability of modern farm and non-farm technologies that take account of relative factor prices and hence in agricultural comparative advantage. Appropriate indicators of agricultural comparative advantage are difficult to assemble, because government policies that distort food markets are so pervasive and because of the range of technologies made available via adaptive research and development (R&D) investments to suit different relative factor scarcities (Hayami & Ruttan 1985; Alston *et al*. 2009*a*,*b*). Thus, the sector's share of national exports relative to the global average, or even net exports as a ratio of exports plus imports of primary agricultural products (both shown in table 1 for the key regions of the world), are rather poor reflections of comparative advantage, and they also conceal much intra-regional diversity.

**Table 1.** Resource endowments and agriculture's share of regional economy, 2000–2006. From World Bank (2008) and (for employment) Sandri *et al*. (2007). n.a., not applicable.

	arable land *per capita* (ha), 2005	fresh water *per capita* ('000 m^3^), 2005	GDP *per capita* (US$), 2005	agriculture's share (%) of GDP, 2006	agriculture's share (%) of employment		agriculture's share (%) of exports, 2006	net agric exports/(agric X + M), 2000–2004
					1960–1964	2000–2004		
world	0.22	6.8	6.6	3	59	44	8	0
HICs	0.36	9.6	31.1	2	17	3	8	0.04
developing countries	0.20	6.3	1.6	10	70	35	11	n.a.
East Asia	0.11	5.0	1.4	12	80	60	8	−0.14
South Asia	0.14	1.2	0.6	18	75	57	13	0.07
Eastern Europe and the CIS	0.57	11.5	3.2	7	n.a.	19	7	−0.06
Middle East and North Africa	0.18	0.8	3.5	12	n.a.	n.a.	5	n.a.
sub-Saharan Africa	0.25	5.1	0.8	15	>80	56	n.a.	0.20
Latin America and the Caribbean	0.27	24.5	4.1	6	48	19	17	0.51

A key determinant of agricultural comparative advantage differences across countries is relative factor endowments, which can change substantially as economies grow at varying rates. Differing technologies also can have an influence on the supply side of the market, and those differences can persist for long periods if governments under-invest in agricultural R&D. As for differences in tastes on the demand side, international diffusion tends to ensure they are far less important than factor endowment differences over the very long term. Nonetheless, changes in the preferred mix of foods away from starchy staples and towards livestock and horticultural products as consumers move from low-income to high-income status can influence comparative advantages within the farm sector.

The simplest model to capture the influence of changes in relative factor endowments in a growing world economy is perhaps that provided by Leamer (1987). His model has just three productive factors: natural resources, labour time and produced capital (human as well as physical, where the human component is defined here to include not only skills but also technologies available in each country). The higher a country's endowment of natural resources relative to the other two factors, when compared with the global average, the stronger its comparative advantage in primary products. The latter can be interpreted as food and agricultural products if the only natural resources are agricultural land and water; but, if a country also has resources that can be depleted through mining (e.g. minerals, energy raw materials or natural forests), then changes in the profitability of such mining also will affect agricultural comparative advantages. Generally, a mining boom, or a sustained inflow of foreign aid, would diminish a country's agricultural comparative advantage (Corden 1984). However, if the boom was driven by a surge in the international price of non-farm tradables (rather than supply driven as with the discovery of a new reserve of minerals or a new mining technology), and the product whose price rose has an agricultural substitute, then producers of that farm product could also benefit—as discussed in §3*a* with respect to biofuels.

Apart from occasional supply-driven mining booms, sustainable economic growth is generally due to growth in produced capital (including available technologies) per worker. Some of any increment in produced capital may be used to expand primary production, but mostly it is used in other sectors. This tendency begins at an earlier stage of development, and thus, at a lower national wage rate, the smaller a country's per-worker endowment of land and other exploitable natural resources, and the smaller its investment in new technologies for agriculture relative to non-farm sectors. Thus, the ranking of countries according to their agricultural comparative advantage is correlated with their farmland/labour endowment ratio, while their capital intensity of agricultural production is correlated with their produced capital/labour endowment ratio. A crude index of the latter is simply *per capita* GDP, reported for 2005 in table 1 along with arable land and fresh water *per capita*.

Global agricultural trade has grown much slower than trade in other products. Prior to the 1960s, farm products accounted for more than 30 per cent of all merchandise trade globally, but since the beginning of this century their share has averaged less than 9 per cent (Sandri *et al*. 2007).

Since agriculture's share of global GDP has also fallen, a more appropriate indicator of the changing extent to which agriculture is globalized is the share of agricultural and food production or consumption that is traded internationally. Table 2 provides estimates of that for various regions, based on a sample of 75 countries that account for all but 1/10 of the world's population and agricultural GDP. Those numbers suggest that agriculture's tradability has increased considerably since the 1960s, rising from about one-ninth to about one-sixth of global production or consumption. However, a glance at the regional data reveals that most of that change is due to increased intra-European trade behind the EU's common external trade barrier, apart from some growth (from low bases) since the 1970s in agricultural imports by Asia and Latin America.

**Table 2.** Export orientation, import dependence and self-sufficiency in global agricultural production, by region,^a^ 1961–2004 (per cent at undistorted prices). From Anderson (2009, ch. 1), compiled from Anderson & Valenzuela (2008) using estimates of total agricultural production valued at undistorted prices and the FAO's total agricultural trade value data for 65 countries that together account for about 90% of the world's population and agricultural GDP.

	1961–1964	1970–1974	1980–1984	1990–1994	2000–2004
*exports as share of production*					
Africa	19	17	12	7	8
Asia	5	4	4	6	5
Latin America	24	27	16	16	27
Western Europe	13	16	27	37	43
USA and Canada	14	14	20	20	21
Australia and New Zealand	41	35	44	43	48
Japan	1	2	1	0	1
all countries	11	11	13	16	16
developing countries	8	8	7	8	8
HICs	14	15	22	26	29
*imports as share of apparent consumption*					
Africa	2	2	5	4	4
Asia	4	4	8	16	14
Latin America	2	4	7	10	17
Western Europe	32	28	34	41	46
USA and Canada	4	4	5	9	12
Australia and New Zealand	3	2	3	5	6
Japan	23	24	24	26	27
all countries	11	10	12	19	18
developing countries	3	4	8	14	13
HICs	18	16	20	25	27
*self-sufficiency ratio*					
Africa	120	117	107	104	105
Asia	102	100	96	89	91
Latin America	129	132	110	107	114
Western Europe	78	85	90	94	94
USA and Canada	111	112	119	114	111
Australia and New Zealand	165	151	174	170	183
Japan	78	78	77	74	74
all countries	100	101	101	96	98
developing countries	105	104	99	93	95
HICs	96	98	103	101	102

^a^Includes intra-EU trade.

Particularly striking is the decline in the extent to which African agricultural production is exported, bringing down the region's agricultural self-sufficiency from 120 per cent to 105 per cent over the four decades to 2000–2004 (table 2). It needs to be kept in mind, though, that this could be in part owing to the region's changing comparative advantages rather than to trade taxes. Such a change in comparative advantage could be because of a boom in other sectors of African economies, for example due to the local discovery, exploitation and exportation of mining products, or because of the large sums of foreign aid flowing into the region, either of which would strengthen a country's currency and thus make its farmers less competitive in international markets. Another possible explanation is the faster growth of farm relative to non-farm productivity in the rest of the world, which is consistent with the relatively slow growth in Africa's crop yields. Alston *et al*. (2009*a*) found that land productivity growth between 1961 and 2005 increased only 2.19 per cent per year in Africa compared with 2.72 per cent in all developing countries, and they note that the lag in farm labour productivity growth was even greater (0.76% for Africa versus 1.93% per year for all developing countries). A third possibility is that other regions have reduced their trade costs, or their anti-agricultural and anti-trade policy biases, more than have countries of sub-Saharan Africa in recent decades. The latter is supported by recently compiled evidence on policy trends reported in Anderson (2009).

### (b) Technological changes and trade costs

In addition to governmental barriers to trade, there are natural trade barriers caused by transport, information and communication costs. Farm products are relatively bulky commodities, making them costly to transport over long distances, especially if they are perishable. Some of them are desired in fresh form, a desire that can be satisfied only in season. Hence, food prices can vary substantially across time and space for these reasons.

If we define globalization as a decline in costs of doing business across space, there has been, and continues to be, great scope for farmers and food consumers to be beneficiaries of its acceleration. When the relevant space includes national borders, a key effect of such cost declines is to enhance the international integration of markets. A standard indicator of such integration is the trade-to-GDP ratio. Merchandise trade for centuries has grown faster than output for all periods (other than between the two world wars), and the gap has been larger in the 1990s than in any earlier period since reliable data became available. According to Maddison (2001, p. 363) merchandise exports as a share of global GDP was only 1 per cent in 1820, 5 per cent in 1870 and 8 per cent in 1913 at 1990 prices. Between 1975–1979 and 2000–2004, however, the share of all goods and services exports as a share of global GDP rose from 19 per cent to 26 per cent (Sandri *et al*. 2007).

The impacts of the drivers of globalization are not uniform across countries, which is showing up in trade specialization data: between 1980–1984 and 2000–2004, the share of non-food manufactures in merchandise exports rose from just over one-quarter to almost two-thirds for middle-income countries (and from less than half to 90% for China), and the share of processed food products in the value of food and agricultural exports over that period rose from 54 per cent to 69 per cent for high-income countries (HICs) and from 49 per cent to 67 per cent for Asia (Sandri *et al*. 2007).

The lowered cost of moving products and people was dominated, in the middle half of the twentieth century, by the falling cost of motor vehicle and aeroplane transportation, thanks to mass production of such goods and associated services. Ocean freight rates (helped by containerization) and telephone charges also fell massively over this period. Transport costs can be crudely captured by the extent to which a product's Cost Insurance and Freight (c.i.f.) import price at its destination port exceeds its Free On Board (f.o.b.) export price at its port of origin. For US merchandise, that markup fell from 10 per cent in the 1950s to 6 per cent in the 1990s (Frankel 2000). An example for agriculture was the change from handling crop products such as grains in bags to bulk for storage and for land and water transportation, reducing substantially transport and storage costs including post-harvest losses. The bag-to-bulk transformation began in industrial countries following World War II and gradually permeated middle-income countries such as Argentina and Brazil, and it is now becoming more widespread in low-income countries too. Other improvements, which need not show up as a reduction in the f.o.b./c.i.f. price gap, are improved transport services such as faster and more frequent schedules and controlled atmosphere containers that allow perishables such as meats, milk products and fresh fruit and vegetables to be transported longer distances by sea or air.

A more recent phenomenon, beginning near the end of the twentieth century, is digital—namely the ICT revolution. Aided by deregulation and privatization of telecom markets in many countries, it has been lowering long-distance communication costs enormously, especially the cost of rapidly accessing and processing knowledge, information and ideas from anywhere in the world. Science has been among the beneficiaries of the digital revolution, spawning yet other revolutions, such as in biotechnology and nanotechnology.

Foreign direct investment (FDI) liberalization sometimes has been a complement to trade liberalization. Developing countries so far are only minor players as hosts of FDI in processed food, beverages and tobacco, however: in 2007, their inflow was less than $3 billion, compared with an inflow of $46 billion into HICs. Flows of FDI into the primary agricultural sector were even less, such that FDI accounted for less than 0.3 per cent of capital formation in developing country agriculture compared with 13 per cent for the overall economy of that country group (UNCTAD 2009, ch. 3). Nonetheless, Reardon & Timmer (2007) argued that FDI has facilitated the transformation of food value chains over the past two decades, in particular via the expansion and merger/takeover activity in supermarket retailing. In most HICs now, no more than five firms account for the majority of sales, and in many of those countries, the four top firms have more than two-thirds of sales.

Supermarkets have been spreading even faster in developing countries than they did in HICs. This is having dramatic effects further up the value chain. First-stage processors, food and beverage manufacturers, and distributors are also becoming more concentrated so as to better match the bargaining power of supermarkets, although typically in narrowly focused industries rather than across the board as in supermarket retailing. Their actions are constrained too by the supermarkets' capacity to develop their own brands and even their own processing and distribution. In turn, these developments are altering dramatically the way farmers are expected to supply those markets, with the emphasis on timely delivery of uniformly high-quality products with very specific attributes (Reardon & Timmer 2007; Swinnen 2007; Reardon *et al*. 2009). According to Swinnen & Vandeplas (2009), though, consumers and possibly even farmers in developing countries are benefitting from the trade and investment liberalization and ICT revolution that have stimulated these changes, because of the fierce competition that ensues among middlemen along the food value chain.

### (c) Agricultural trade distortions and policy reforms

In addition to agricultural trade being affected by economic growth and declining trade costs, it has been greatly affected by distortionary government policies. Since the 1950s, world agriculture has been characterized by the persistence of high agricultural protection in developed countries, by anti-agricultural and anti-trade policies of developing countries and by the tendency for both sets of countries to use trade measures to stabilize their domestic food market—thereby exacerbating price fluctuations in the international marketplace. This disarray has not only been highly inefficient but has also contributed to global inequality and poverty (since the vast majority of the world's poorest households depend directly or indirectly on farming for their livelihoods; see Anderson *et al.* 2010*a*). The situation worsened up to the mid-1980s, with agricultural protection in Europe, North America and Japan peaking and international food prices plummeting in 1986, thanks in large measure to an agricultural export subsidy war between the US and the European community. Meanwhile, many developing countries had been reducing farm incomes not only by heavily taxing agricultural exports but also, albeit indirectly, by protecting manufacturers from import competition and overvaluing the national currency.

**Figure 1.**
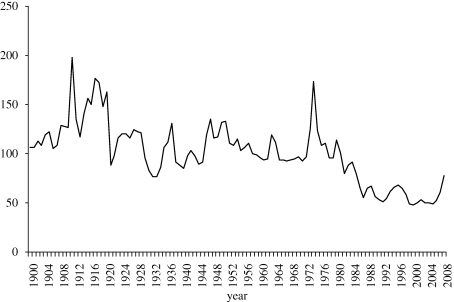
Real international food price index, 1900–2008 (1977–1979 = 100). The deflator used is the price of manufactured exports to developing countries from the five largest HICs (France, Germany, Japan, the UK and the USA). Author's compilation using data from Pfaffenzeller *et al*. (2007), updated from 2004 with data from www.worldbank.org/prospects. Solid line, real food price index.

This disarray in world agriculture meant that there was over-production of farm products in HICs and under-production in more-needy developing countries. It also meant there was less international trade in farm products than would be the case under free trade, thereby ‘thinning’ the market for these weather-dependent products and thus making them more volatile. The extent of that volatility is evident in figure 1. Using a stochastic model of world food markets, one study estimates that the coefficient of variation of international food prices in the 1980s was three times greater than it would have been under free trade and that the volume of international trade in grains, livestock products and sugar was half what it could have been (Tyers & Anderson 1992, tables 6.9 and 6.14).

During the past quarter-century, numerous developing countries and HICs have begun to reform their agricultural price and trade policies. This has contributed to the rise in the extent to which farm products are traded internationally, noted above. Much of this reform was undertaken unilaterally or as part of regional trading arrangements, but some was also undertaken in response to international pressures such as Uruguay Round stipulations, commitments required for accession to the World Trade Organization (WTO) and structural adjustment loan conditionality by international financial institutions. Meanwhile, reforms in some middle-income economies (most noticeably Korea) have ‘overshot’, going from discouraging their farmers to protecting them from import competition—which raises concerns that other emerging economies may follow suit and pursue the same agricultural protection growth path of more-advanced economies in earlier stages of their economic development.

A recent World Bank research project (see Anderson (2009) and www.worldbank.org/agdistortions) developed a series of indicators to measure the impact of those interventions and subsequent policy developments on farmers' incentives. Its most basic measure, the nominal rate of assistance (NRA) is the percentage by which government policies have raised gross returns to farmers above what they would be without the government's intervention (or lowered them, if the NRA is negative). Farmers are affected not just by prices of their own outputs but also (albeit indirectly through changes to factor market prices and the exchange rate) by the incentives offered to non-agricultural producers. That is, it is *relative* prices and hence *relative* rates of government assistance that affect producers' incentives, so a relative rate of assistance (RRA) was also calculated.

**Figure 2.**
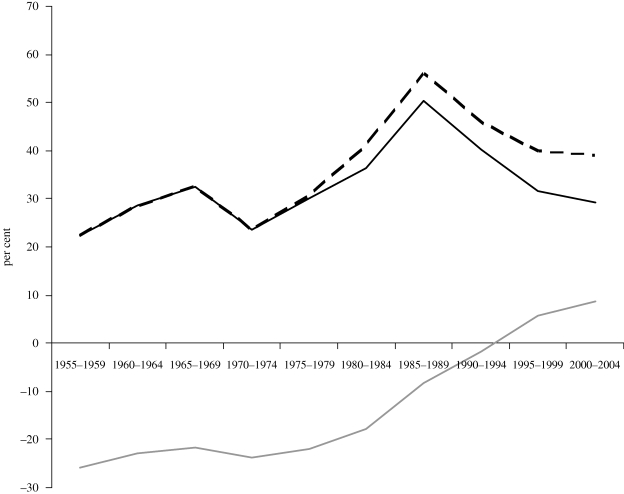
Nominal rates of assistance to agriculture in HICs and European transition economies and in developing countries, 1955–2004 (per cent, weighted averages). The European transition economies is denoted by the World Bank as ECA, its acronym for (Central and Eastern) Europe and Central Asia. From Anderson (2009, ch. 1), based on estimates in Anderson & Valenzuela (2008). Black line, HIC and ECA; dashed line, HIC and ECA, including decoupled payments; grey line, developing countries.

The NRAs from the World Bank study, which involves 75 countries (including 20 HICs) which together account for 92 per cent of global agricultural GDP, are sumarized in figure 2. They reveal that assistance to farmers in HICs rose steadily from the mid-1950s until the end of the 1980s, apart from a small dip when international food prices (see figure 1) spiked around 1973–1974. After peaking at more than 50 per cent in the mid-1980s, the average NRA for HICs has fallen a little, depending on the extent to which one believes that some new farm programmes are ‘decoupled’ in the sense of no longer influencing production decisions. For developing countries, the average NRA for agriculture has been rising, but from a level of around −25 per cent during the period from the mid-1950s to the early 1980s to nearly 10 per cent in the first half of the present decade.

**Figure 3.**
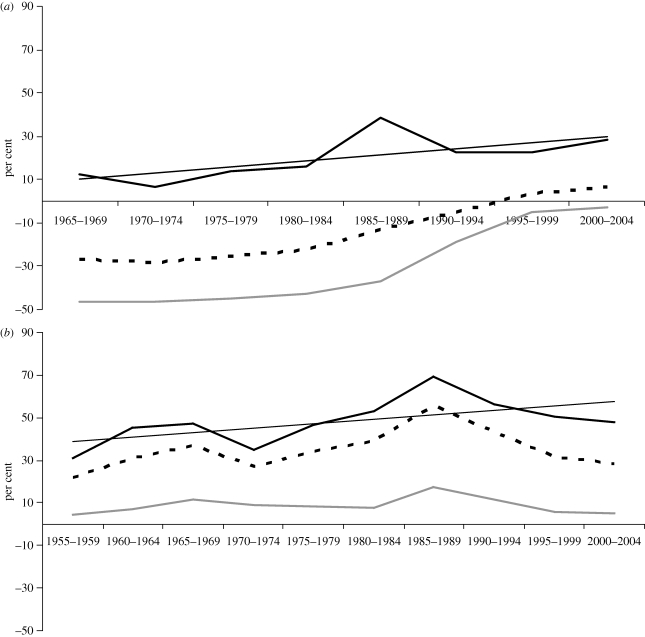
Nominal rates of assistance to exportable, import-competing and all covered agricultural products (covered products only, and the total also includes non-tradables), HICs and developing countries, 1955–2007. (*a*) Developing countries. (*b*) HICs plus Europe's transition economies. From Anderson (2009, ch. 1), based on estimates in Anderson & Valenzuela (2008). Black lines, import competing; grey lines, exportables; dashed lines, total.

The average NRA for developing countries conceals the fact that the exporting and import-competing subsectors of agriculture have very different NRAs. Figure 3 reveals that while the average NRA for exporters has been negative throughout (going from −20% to −30% before coming back up to almost zero in 2000–2004), the NRA for import-competing farmers in developing countries has fluctuated between 20 per cent and 30 per cent (and even reached 40% in the years of low prices in the mid-1980s). The anti-trade bias within agriculture (the taxing of both exports and imports) has diminished for developing countries since the mid-1980s, but the NRA gap between the import-competing and export subsectors still averages around 20 percentage points (and it has grown to 40 percentage points for HICs, although there even exporters have enjoyed positive NRAs). Figure 3 also reveals that the NRA for import-competing farmers in developing countries has increased at virtually the same pace as that in HICs, suggesting that growth in agricultural protection from import competition is something that tends to begin at modest levels of *per capita* income rather than being a phenomenon exclusive to HICs.

**Figure 4.**
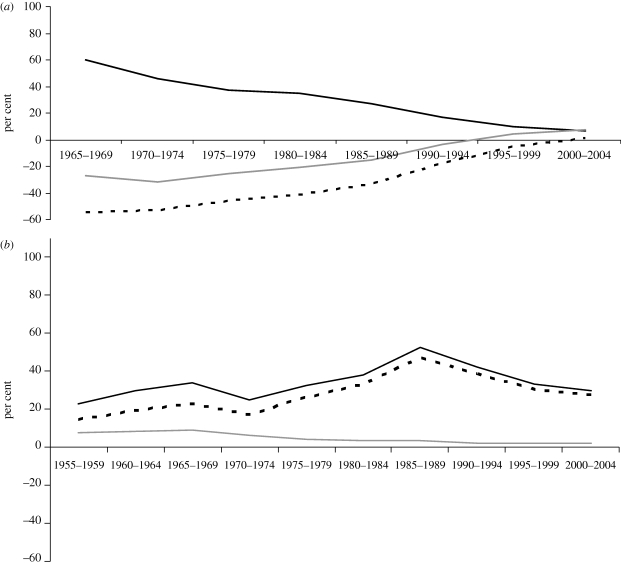
Nominal rates of assistance to agricultural and non-agricultural sectors and relative rate of assistance, developing countries and HICs, 1955–2004 (per cent, production-weighted averages across countries). (*a*) Developing countries. Dashed line, RRA; black line, NRA non-agricultural tradables; grey line, NRA agricultural tradables. (*b*) HICs. Black line, NRA agriculture; grey line, NRA non-agriculture; dashed line, RRA. The RRA is defined as 100 * [(100 + NRAag^t^)/(100 + NRAnonag^t^) − 1], where NRAag^t^ and NRAnonag^t^ are the percentage NRAs for the tradable parts of the agricultural and non-agricultural sectors, respectively. From Anderson (2009, ch. 1), based on the estimates in Anderson & Valenzuela (2008).

The improvement in farmers' incentives in developing countries is understated by the above NRA estimates, because those countries have also reduced their assistance to producers of non-agricultural tradable goods, most notably via cuts in restrictions on imports of manufactures. The decline in the weighted average NRA for the latter, depicted in figure 4, was clearly much greater than the increase in the average NRA for tradable agricultural sectors for the period to the mid-1980s, consistent with the finding of Krueger *et al*. (1988, 1991) two decades ago. For the period since the mid-1980s, changes in the NRAs of both sectors have contributed almost equally to the improvement in incentives to farmers. The RRA for developing countries as a group went from −46 per cent in the second half of the 1970s to 1 per cent in the first half of the present decade. This increase (from a coefficient of 0.54 to 1.01) is equivalent to an almost doubling in the relative price of farm products, which is a huge change in the fortunes of developing country farmers in just a generation. This is mostly because of the changes in Asia, but even for Latin America this relative price hike is one-half, while for Africa this indicator improves by only one-eighth. As for HICs, assistance to manufacturing was on average much less than assistance to farmers, even in the 1950s, and its decline since then has had only a minor impact on that group's average RRA (figure 4). The exceptions are Australia and New Zealand, where manufacturing protection had been very high and its decline occurred several decades later than in other HICs (Anderson *et al*. 2007).

The above influences of policies focus on long-term trends, but policies also influence year-to-year fluctuations around trend prices and quantities as governments seek to reduce fluctuations in domestic food markets. One way for a country to achieve that objective is by varying the restrictions on its international trade in food according to seasonal conditions domestically and changes in prices internationally. Anderson *et al*. (2010*b*) capture this phenomenon by estimating the elasticities of transmission of the international product price to the domestic market, using a geometric lag formulation for each product for all focus countries for the period since 1985. The unweighted average estimate for the short-term elasticity for 12 key products is 0.54, suggesting that within the first year little more than half the movement in international prices of those farm products is transmitted domestically.

To assess how far the world had come, and how far it still has to go, in rectifying the disarray in world agriculture, Valenzuela *et al*. (2009) use the World Bank's global economy-wide model known as Linkage to provide a combined retrospective and prospective analysis. It quantifies the impacts both of past reforms and current policies by comparing the effects of the recent World Bank project's distortion estimates for the period 1980–1984 with those of 2004. The findings from that economy-wide modelling study suggest that:

— Policy reforms from the early 1980s to the mid-2000s improved global economic welfare by US$233 billion per year, and removing all goods market distortions that remained in 2004 would add another US$168 billion per year (in 2004 US dollars) implying, in terms of global welfare, that the world had moved three-fifths of the way towards global free trade in goods over that quarter-century.
— Developing economies benefitted proportionately more than high-income economies (1.0% compared with 0.7% of national income) from those past policy reforms and would gain nearly twice as much as HICs if all countries were to complete that reform process (an average increase of 0.9% compared with 0.5% for HICs).
— Of those prospective welfare gains from global goods trade liberalization, 70 per cent would come from agriculture and food policy reform, which is a striking result given that the shares of agriculture and food in global GDP and global trade are only 3 per cent and 6 per cent, respectively.
— If the policies distorting goods trade in 2004 were removed, the share of global production of farm products that is exported would rise from 8 per cent to 13 per cent (excluding intra-EU trade), thereby reducing instability of prices and reducing the quantities of those products traded.
— The developing countries' share of the world's primary agricultural exports rose from 43 per cent to 55 per cent, and its share of farm output from 58 per cent to 62 per cent, because of the reforms since the early 1980s, and removing the remaining goods market distortions would boost their export and output shares even further, to 64 per cent and 65 per cent, respectively.
— For developing countries as a group, net farm income (value added in agriculture) is estimated to be 4.9 per cent higher than it would have been without the reforms of the past quarter-century, and if the farm price and trade policies remaining in 2004 were removed, then net farm incomes in developing countries would rise a further 5.6 per cent, compared with just 1.9 per cent for non-agricultural value added.

## 3. Future drivers and uncertainties to 2050

With this as background, we are now able to consider the likely drivers of changes in national agricultural comparative advantages, trade costs and pertinent policies over the next four decades and their associated uncertainties and impacts on global farm trade. The list includes the following, each of which is considered in turn in the rest of this section of the paper:

— growth in population, incomes and farm productivity;
— crude oil price trends and fluctuations and their impact on biofuel demand;
— trade costs, the supermarket revolution and related changes in food value chains;
— developments in policies distorting agricultural incentives;
— climate changes and national and global policy responses;
— reforms to water institutions and policies; and
— changes in agricultural R&D investments in response to the above.

### (a) Population, income and productivity growth rates

The economic recession in the USA and Europe since 2007 has slowed global economic growth. How long the recovery will take is uncertain because it depends on how quickly risk perceptions abate, which depends in turn on on-going government macroeconomic and trade policy responses (McKibbin & Stoeckel 2009). In that process of readjustment, while long-term growth rates to 2050 may not be greatly affected, currencies may be realigned in ways that have long-term effects on comparative advantages in farm products. However, there is too much uncertainty surrounding such possibilities at this stage to do more than simply note them.

One recent set of population and *per capita* income growth projections to 2050 is summarized in table 3. Clearly, these projections imply significant changes to the economic centres of gravity of consumption in the global economy, given differing income elasticities of demand for various products. They also affect the supply side of each economy: population growth along with demographic changes and labour–leisure choices influence the growth of the workforce, and *per capita* income growth suggests an expansion in the endowment of capital, whether it be in the form of physical assets, workforce skills or new technologies.

**Table 3.** Global population and GDP *per capita* by region, actual 2005 and projected 2050. From Medvedev & van der Mensbrugghe (2008).

	population (billion)		real GDP *per capita* (2005 US$ '000)		real GDP *per capita* (% of global average)	
	2005	2050^a^	2005	2050	2005	2050
world	6.4	8.8 (9.1)	6.6	15.1	100	100
HICs	1.1	1.1 (1.2)	31.1	58.3	470	385
developing countries	5.3	7.7 (7.9)	1.6	9.1	25	60
East Asia	1.9	2.3 (2.2)	1.4	12.8	21	84
South Asia	1.5	2.3 (2.3)	0.6	4.7	9	31
Eastern Europe and CIS	0.4	0.4 (0.7)	3.2	23.6	49	156
Middle East and North Africa	0.3	0.6 (0.6)	3.5	6.5	53	43
sub-Saharan Africa	0.7	1.4 (1.7)	0.8	4.8	11	32
Latin America and Caribbean	0.6	0.8 (0.7)	4.1	13.8	62	90

^a^Alternative population projections from the FAO are shown in parentheses (from Fischer *et al.* 2009).

In economy-wide computable general equilibrium model projections, it is common to represent physical capital assets and human skills explicitly, but to incorporate new technologies simply as shocks to total factor productivity (TFP; the number of units of each input needed to produce a unit of output). The latter can be determined endogenously if the modeller accepts projections of growth in *per capita* income and in the various factors of production, but it is then a challenge to allocate that aggregate TFP shock to different sectors and to different industries within those sectors. Typically, the agricultural sector's TFP growth rate is assumed to exceed that for the rest of the economy, based on historical experience (see Martin & Mitra 2001), so as to ensure the relative price of farm products declines over time as in the second half of the twentieth century (see figure 1). With the growth in international food prices over the 2003–2008 period, however, expectations about their future trend are now less certain. Is that rise just due to a rundown of grain stocks globally, or is it also because of the greater neglect of public investment on agricultural R&D in recent decades (Alston *et al*. 2009*b*; Royal Society 2009)? The possibilities of technological catch-up by lagging regions through faster international technology transfer also need to be considered (e.g. via the Green Revolution for Africa initiative of the Gates and Rockefeller Foundations, but also bearing in mind the apparent recent surge in inflow of FDI in farming from countries relatively poorly endowed with farm land and water; see von Braun & Meinzen-Dick 2009). This suggests that more than one set of assumptions about productivity growth is needed in developing a family of baselines for projections of agricultural productivity to 2050.

Also of more relevance to projections now than in the past are assumptions about food consumption growth. Previously, modellers have relied on past econometric evidence, suggesting that price and income elasticities of demand for food decline with *per capita* income, and earlier for lower-valued foods such as staple grains and tubers than for livestock and horticultural products. The latter switch will be especially important with the rapid income growth in populous emerging economies such as Brazil, China and India. However, consumer concerns for food quality, food safety and the environment also need to be considered, especially for HICs. Environmental concerns affect things such as the disposal of packaging or the carbon footprint associated with the transport of goods and hence a desire to ‘buy local’ or at least to know of the country of origin. Increasing numbers of consumers wish to know how products are produced on-farm and processed, so as to assess whether they are causing environmental damage or reducing animal welfare. The continuing preference of some consumers to avoid foods containing genetically modified organisms (GMOs) is a clear case in point (Qaim 2009). This consumer concern has already led to significant government barriers to trade based on production processes and to constraints on domestic production. If that behaviour persists, models of international trade need to differentiate between products that may or do not contain GMOs. Now that traceability information along with other attributes can be stored on barcodes, these and related biosecurity concerns can be reflected in the demands that the large supermarket chains place on their suppliers for information on myriad attributes of products. This is adding to the need to incorporate greater agricultural product differentiation across suppliers in trade models.

It could be argued that the above concerns of consumers are confined to HICs, especially Western Europe and Japan, where the quantity of food consumed is unlikely to grow rapidly over the next four decades because of relatively low population and income growth and low-income elasticities of demand for farm products there. However, that would be to miss the point that high-income consumers are willing to pay substantial premia for foods that are perceived to be safer, of higher quality and produced with minimal damage to the environment and animal welfare. They are thus potentially highly profitable markets to which all farmers seek access, including those in developing countries—notwithstanding the disadvantage due to their higher carbon footprint insofar as more transportation is probably required to get their produce to those northern markets than is the case for local import-competing farmers.

### (b) Crude oil price trends: effect on biofuel demand

While the real price of crude oil spiked briefly in mid-2008 at nearly three times its previous record, it provides no guidance as to the long-term trend price of petroleum and other energy raw materials. Spikes in the spot price can occur whenever there is a sudden change in expectations (including about OPEC cartel actions), given the low short-term price elasticities of demand and supply for crude oil. Long-term trend prices, on the other hand, are affected by government taxes and developments in known reserves and in demand, which tend to change relatively slowly as economies grow. Technological innovations in exploration and exploitation have caused reserves to expand faster than demand, so the world is apparently not running out of fossil fuels: according to Smith (2009), the ratio of reserves to annual production of crude oil has grown from a multiple of 29 years in 1980 to 45 years in 2008, and if unconventional petroleum resources (heavy oil, oil sands and oil shale) are included, that adds another 160 years of available supplies at current consumption levels.

The capacity of petroleum prices to spike occasionally is not unlike that for grains. As Wright (2009) pointed out, wheat, rice and maize are highly substitutable in the global market for calories, and when aggregate stocks decline to minimal feasible levels for trading and processing, prices become highly sensitive to small shocks. By the middle of the past decade, grain stocks-to-use ratios had declined to their lowest levels for 25 years due to high-income growth in emerging economies and de-stocking in China (Wiggins & Keats 2010). When there were then some crop failures plus a surge in demand because of biofuel mandates and subsidies, grain prices started rising. The crude oil price spike in 2008 raised further the demand for biofuels (as well as fuel and fertilizer input costs for farmers), and a sequence of trade restrictions by key grain exporters, beginning in the thin global rice market in the autumn of 2007, led to panic buying.

The linkage between crude oil and food prices will remain strong when petroleum prices exceed the threshold that makes biofuel production privately profitable on a significant scale, as in 2005–2008 (FAO 2008; IMF 2008; DEFRA 2010; Pfuderer *et al*. 2010). A continuation of biofuel subsidies and mandates will make this co-movement in above-trend prices more common, as will the development of new biofuel crop production technologies that effectively lower the threshold oil price above which ethanol or biodiesel production is profitable (Chakravorty *et al*. 2009; Rajagopal *et al*. 2009). The latter has considerable potential over the next four decades, especially if private life science companies view investments in biofuel crop R&D as more profitable than R&D in politically sensitive GM food crops.

Mandates to include an increasing proportion of biofuels in road transport fuel are now in place in most OECD countries and in Brazil. The current targets in the EU mandate go through to 2020, and those of the US to 2022. These policy measures, if they continue and remain inflexible, will add a certain demand for biofuel crops no matter what happens to fossil fuel and food prices. This will not reduce the extent of any downward food price spike, however, because biofuel production will be privately profitable and so the mandates will tend to be redundant when grain and oilseed prices are very low relative to fossil fuels prices. On the other hand, mandates will exacerbate the extent of any upward food price spike, because fuel retailers will be required to include in their road fuel mix at least the mandated quantity of biofuel regardless of its high cost.

### (c) Trade costs, the supermarket revolution and related changes in food value chains

The ICT revolution will continue to lower trade costs, including for supermarkets as they search globally for the lowest-cost suppliers of products with the attributes desired by their customers. Such searching by supermarkets will increase also in response to governments lowering the remaining barriers to FDI in retailing and associated logistics services. This will more or less offset the impact of any new carbon taxes or their equivalent on transportation costs. The consequences of a continuing supermarket revolution will spread right along the food value chain. One is that first-stage processors, food and beverage manufacturers, and distributors will become more concentrated so as to better match the bargaining power of supermarkets. Even so, supermarkets will exploit their capacity to develop their own brands and even their own processing and distribution. In turn, these developments will alter dramatically the way farmers supply those markets, with the emphasis on timely delivery of uniform-quality products leading to more-efficient (possibly larger) farmers displacing less-efficient ones and thereby raising agricultural productivity growth. Insofar as large supermarkets in HICs source also from farmers in other countries, their private standards will be set with at least some consideration to the costs they impose on foreign suppliers, and so may be less trade restricting than they would be without that feature of globalization.

### (d) Policies distorting agricultural incentives

The reasons why some countries have reformed their price-distorting agricultural and trade policies more than others in recent decades provide hints as to what to expect in coming decades. The reasons are varied. Some countries reformed unilaterally, apparently having become convinced that it is in their own national interest to do so. China is the most dramatic and significant example of the past three decades among developing countries, and Australia and New Zealand among the HICs (Anderson 2009). Other developing countries may have done so partly to secure bigger and better loans from international financial institutions and then, having taken that first step, they have continued the process, even if somewhat intermittently. India is one example, but there are numerous other examples in Africa and Latin America. And some countries have reduced their agricultural subsidies and import barriers at least partly in response to the General Agreement on Tariffs and Trade's multilateral Uruguay Round Agreement on Agriculture and to opportunities to form or expand regional integration agreements. The EU is the most important example of committing to reductions in farm protection, helped by its desire for otherwise costly preferential trade agreements including its expansion eastwards.

The EU reforms suggest that growth in agricultural protection can be slowed and even reversed if accompanied by re-instrumentation away from price supports to decoupled measures or more direct forms of farm income support—but the wealthiest Western European countries (Norway and Switzerland), like Japan, continue to resist external pressure to undertake major reform. The stark example of Australia shows that one-off buyouts can bring faster and even complete reform. In the USA, by contrast, most subsidy cuts in the 1990s proved to be short lived and have since been reversed, with one set of analysts seeing few signs of that changing in the foreseeable future (Orden *et al*. 2010).

In the developing countries, where levels of agricultural protection are generally below those in HICs, there are fewer signs of a slowdown of the upward trend in protection from agricultural import competition over the past half-century. Indeed, there are numerous signs that the governments of developing countries want to keep open their options to raise agricultural NRAs in the future, particularly via import restrictions. One indicator is the high tariff bindings to which developing countries committed themselves following the Uruguay Round (Anderson & Martin 2006, table 1.2). Another is the demand by many developing countries to be allowed to maintain their rates of agricultural protection from import competition for reasons of food security, livelihood security and rural development. This view has succeeded in bringing ‘special products’ and a ‘special safeguard mechanism’ into the multilateral trading system's agricultural negotiations, even though such policies would raise domestic food prices in developing countries and thus may worsen poverty and food security of the urban poor while exacerbating instability in international markets for farm products.

If the WTO's Doha Development Agenda collapses, or if Doha leads to only a weak agricultural agreement full of exceptions for politically sensitive products and safeguards, the governments of HICs may find it more difficult to ward off agricultural protection lobbies. This would make it more likely that developing countries choose an agricultural protection path. The potential cost of this alternative counterfactual could be several times the estimated benefit of a successful Doha agreement when the counterfactual is assumed to be current policies (Bouët & Laborde 2008). Regional and other preferential trading arrangements may be able to reduce farm protection growth somewhat, but the experiences with regional integration arrangements to date is mixed.

### (e) Climate change and policy responses

Effects of climate change on aggregate global agricultural production and its location across countries and regions without and with mitigation and adaptation are great unknowns, not least because there are many possible government policy responses unilaterally and multilaterally. Moreover, the uncertainties about what policy instruments will be adopted by whom and when will be spread over decades rather than just the next few years. Land use undoubtedly will be affected non-trivially; carbon credits and emissions trading will have unknown and possibly major effects depending among other things on whether/how/when agriculture and forestry are included in the schemes of various countries, as will any border tax adjustments or other sanctions imposed on imports from countries deemed to be not sharing the burden of reducing greenhouse gases; biofuel mandates and subsidies and emerging biofuel crop technologies are likely to increasingly affect food markets, and even more so if carbon taxes or emission caps raise the user price of fossil fuels; crop yield fluctuations will be greater because of weather volatility and especially more extreme weather events, leading to further triggers for trade policy interventions aimed at stabilizing domestic food markets and so on.

The literature on these and myriad other ways in which agricultural markets are expected to be affected directly and indirectly by climate change and associated policy and technological responses are growing exponentially. Numerous global economic modellers have begun analysing the possible effects of some of the above influences on the international location of agricultural production and trade in particular. One of the more widely cited is Cline (2007), who predicted that by the 2080s, even with carbon fertilization, agricultural output will be 8 per cent lower in developing countries, 8 per cent higher in HICs and 3 per cent lower globally. However, mitigation policies could have an adverse effect on industrialization in developing countries and lead to their agricultural sector in aggregate benefitting indirectly, although different types of border tax adjustments by HICs would affect the outcome non-trivially (Mattoo *et al*. 2009*a*,*b*). It is clearly very difficult to discern what the main influences are likely to be over the next four decades, let alone to quantify the effects of even the most likely of them. This underscores the need for sensitivity analysis around any baseline scenario to 2050 that does not include any of the influences listed in the previous paragraph.

### (f) Reforms to water institutions and policies

Water is essential for growing food and critical for food security, but in many parts of the world it has been one of the most-abundant factors of production used in agriculture. Certainly, it is not evenly spread across the world (see column 2 of table 1), and irrigation water property rights and water markets are poorly developed in most countries.

With population growth and the increasing need for non-farm uses of water, the urgency for policy reform in this area is growing, especially outside temperate, well-watered areas such as Europe (Rosegrant *et al*. 2002). The experiences with reforms to date, such as in the USA and Australia, indicate there will be much trial and error in policy design and implementation and it will take many decades before water markets are as efficient as farm land markets. This suggests that irrigation water costs could well rise in coming decades but to varying extents across the globe and in ways that could have non-trivial impacts on the optimal location of certain water-intensive crops.

### (g) Agricultural research and development investments

Agricultural R&D investments have had a huge payoff (Alston *et al*. 2000). Yet there has been a considerable slowdown in such investments over the past two decades, and this may already be contributing to a slowing of agricultural productivity growth (Alston *et al*. 2009*a*,*b*). If that slowdown in investment was in response to the low prices of food in international markets in the mid-1980s, then the rise in those prices in recent years, together with the newly perceived need for adaptive research in response to climate change and increased water scarcity, may boost farm productivity growth over the next four decades. Advances in biotechnology will help raise potential yields in field trials and thus attainable yields in the best farms, but much can also be gained by reducing the gap between those attainable yields and average on-farm yields, particularly in developing countries.

Part of the slowdown in traditionally measured gains from agricultural research in recent decades may be due to research being directed away from things such as maintaining and improving yields and towards conservation of natural resources and the environment. It is likely that climate change concerns will also lead to some re-direction of R&D investment, to goals such as crop tolerance to drought and other extreme weather events.

Another large dilemma for research administrators, both public and private, is how much effort to direct to transgenic foods. While there remains strong opposition by some consumers and governments of large countries to GM food production and imports, the returns from such research will be dampened, both absolutely and relative to efforts to produce non-food GM crops (cotton, biofuels and other industrial crops). R&D on the latter will reduce the upward pressure that demands for those non-food crops would otherwise put on food prices, but the anti-GM food stance will continue to reduce the potential for biotechnology to lower food prices in countries where GM food is discouraged or banned—with major implications for bilateral trade flows since it effectively divides the world food supplies into two separate markets (Anderson & Jackson 2006).

## 4. Conclusions and policy implications

Recent globalization has been characterized by a decline in the costs of cross-border trade in farm and other products. It has been driven by the ICT revolution, declines in real transport costs and—in the case of farm products—by reductions in governmental distortions to agricultural incentives and trade. The first but maybe not the second of these drivers will continue in coming decades. World food prices will depend also on whether/by how much farm productivity growth continues to outpace demand growth. Demand in turn will be driven not only by population and income growth, but also by crude oil prices if they remain at current historically high levels, since that will affect the biofuel demand. Climate change mitigation policies and adaptation, water market developments and market access standards including for transgenic foods add to future agricultural production, price and trade uncertainties.

The key issues that modellers need to grapple with in projecting world agricultural markets to 2050—assuming they have already dealt with simulating the macro-policy settings and the evolving pattern of international capital flows and their effects on currency exchange rates and broad comparative advantages—are what to assume about trends and fluctuations for each country and hence globally in:

— price- and trade-distorting sectoral policies that alter farmer and consumer incentives (which in turn depend on the outcome of on-going Doha trade negotiations and any subsequent WTO rounds and regional trading agreements);
— TFP growth on farms in GM-free and GM-tolerant country settings;
— petroleum and related fossil fuel prices and their impact on biofuel crop productivity growth; and
— climate variables and policy responses to climate change, including for water and biofuels.

Governments can do, and some already are doing, things to reduce the uncertainties associated with the above issues. First, WTO members are trying to conclude the Doha trade negotiations. Trade opening can lead to more effective resource conservation, improve global welfare and reduce inequality, poverty, malnutrition and hunger (Anderson *et al.* 2010*a*). The signs are not promising for a very ambitious outcome from Doha, however. It is even possible that exceptions for ‘sensitive’ and ‘special’ agricultural products and a special safeguard mechanism to protect developing countries from import surges could discount heavily the value of any new commitments. In that case, and perhaps even more so if WTO members fail to reach a conclusion to the Doha round, agricultural protection growth could resume in HICs and/or be emulated in developing countries, with both country groups varying their protection rates in an attempt to stabilize their domestic market—but at the expense of destabilizing international food markets and thereby encouraging even more countries to thus intervene at their national border.

Second, governments could commit to a more ambitious programme of support for agricultural R&D investment, so as to slow or reverse the decline since the 1990s in such investments. Lags between R&D investments and farm productivity growth are very long, but certainly results would show within the next four decades. Governments yet to embrace the relatively new agricultural biotechnologies could reassess their stance in the light of (i) the experiences of countries that have accepted this technology (as environmental effects have been mostly benign or positive and no food safety issues are evident) and (ii) the higher benefits from expanding such investments now that food price levels are higher and climate changes are requiring farmer adaptation.

Finally, governments could make clear what their policy responses will be to climate change. The difficulties associated with this global issue make multilateral trade negotiations look easy, as was clearly demonstrated by the difficulty in drafting a communiqué at the end of the Copenhagen global conference on the issue in December 2009.

## References

[RSTB20100131C1] Alston J. M. Marra M. C. Pardey P. G. Wyatt T. J. 2000 A meta-analysis of rates of return to agricultural R&D: Ex Pede Herculem? Washington, DC: International Food Policy Research Institute

[RSTB20100131C2] Alston J. M. Beddow J. M. Pardey P. G. 2009 *a* Mendel versus Malthus: research, productivity and food prices in the long run. Staff Paper P09-1. Department of Applied Economics, University of Minnesota, St Paul, MN, revised September 10.1126/science.1170451 19729642

[RSTB20100131C3] Alston J. M. Beddow J. M. Pardey P. G. 2009 *b* Agricultural research, productivity, and food prices in the long run. Science 325, 1209–1210 (doi:10.1126/science.1170451) 19729642 10.1126/science.1170451

[RSTB20100131C4] Anderson K. 1987 On why agriculture declines with economic growth. Agric. Econ. 1, 195–207 (doi:10.1016/0169-5150(87)90001-6)

[RSTB20100131C5] Anderson K. (ed.) 2009 Distortions to agricultural incentives: a global perspective, 1955–2007. London, UK: Palgrave Macmillan; Washington, DC: World Bank

[RSTB20100131C6] Anderson K. Jackson L. A. 2006 Transgenic crops, EU precaution, and developing countries. Int. J. Technol. Glob. 2, 65–80

[RSTB20100131C7] Anderson K. Martin W. (eds) 2006 Agricultural trade reform and the Doha development agenda. London, UK: Palgrave Macmillan; Washington, DC: World Bank

[RSTB20100131C8] Anderson K. Valenzuela E. 2008 Global estimates of distortions to agricultural incentives, 1955–2007. Spreadsheet available at www.worldbank.org/agdistortions

[RSTB20100131C9] Anderson K. Lloyd P. J. MacLaren D. 2007 Distortions to agricultural incentives in Australia since World War II. Econ. Rec. 83, 461–482

[RSTB20100131C48] Anderson K. Cockburn J. Martin W. (eds) 2010 *a* Agricultural price distortions, inequality and poverty. Washington, DC: World Bank

[RSTB20100131C10] Anderson K. Croser J. Sandri D. Valenzuela E. 2010 *b* Agricultural distortion patterns since the 1950s: what needs explaining? In The political economy of agricultural price distortions, ch. 2 (ed. Anderson K.). Cambridge, UK: Cambridge University Press

[RSTB20100131C11] Bouët A. Laborde D. 2008 The potential cost of a failed Doha round. IFPRI Issue Brief 56, International Food Policy Research Institute, Washington, DC, December

[RSTB20100131C12] Chakravorty U. Hubert M.-H. Nostbakken L. 2009 Fuel versus food. Annu. Rev. Resour. Econ. 1, 645–663 (doi:10.1146/annurev.resource.050708.144200)

[RSTB20100131C13] Cline W. R. 2007 Global warming and agriculture: impact estimates by country. Washington, DC: Center for Global Development and Peterson Institute for International Economics

[RSTB20100131C14] Corden W. M. 1984 Booming sector and Dutch disease economics: survey and consolidation. Oxf. Econ. Pap. 36, 359–380

[RSTB20100131C15] DEFRA 2010 The 2007/08 agricultural price spikes: causes and policy implications. London: DEFRA, HM Government

[RSTB20100131C16] FAO 2008 The state of food and agriculture: biofuel prospects, risks and opportunities. Rome, Italy: FAO

[RSTB20100131C49] Fischer R. A. Byerlee D. Edmeades G. O. 2009 Can technology deliver on the yield challenge to 2050? Paper for an Expert Meeting on How to feed the World in 2050, Food and Agriculture Organization, Rome, Italy, 24–26 June.

[RSTB20100131C17] Frankel J. 2000 Globalization of the economy. In Governance in a Globalizing World, ch. 2 (eds Nye J. S. Jr Donahue J. D.). Washington, DC: Brookings Institution Press

[RSTB20100131C18] Hayami Y. Ruttan V. W. 1985 Agricultural development: an international perspective, revised edn Baltimore, MD: Johns Hopkins University Press

[RSTB20100131C19] IMF 2008 Food and fuel prices: recent developments, macroeconomic impact, and policy response. Washington, DC: IMF

[RSTB20100131C20] Krueger A. O. Schiff M. Valdés A. 1988 Measuring the impact of sector-specific and economy-wide policies on agricultural incentives in LDCs. World Bank Econ. Rev. 2, 255–272 (doi:10.1093/wber/2.3.255)

[RSTB20100131C21] Krueger A. O. Schiff M. Valdés A. 1991 The political economy of agricultural pricing policy, volume 1: Latin America, volume 2: Asia, and volume 3: Africa and the Mediterranean. Baltimore, MD: John Hopkins University Press for the World Bank

[RSTB20100131C22] Leamer E. E. 1987 Paths of development in the three-factor, n-good general equilibrium model. J. Polit. Econ. 95, 961–999 (doi:10.1086/261498)

[RSTB20100131C23] Maddison A. 2001 The world economy: a millennium perspective. Paris: Development Centre Studies, OECD

[RSTB20100131C24] Martin W. Mitra D. 2001 Productivity growth and convergence in agriculture and manufacturing. Econ. Dev. Cultur. Change 49, 403–423 (doi:10.1086/452509)

[RSTB20100131C25] Mattoo A. Subramanian A. van der Mmensbrugge D. He J. 2009 *a* Can global de-carbonization inhibit developing country industrialization? Policy Research Working Paper 5121, World Bank, Washington, DC, November

[RSTB20100131C26] Mattoo A. Subramanian A. van der Mmensbrugge D. He J. 2009 *b* *Reconciling climate change and trade policy*. Policy Research Working Paper 5123, World Bank, Washington, DC, November

[RSTB20100131C27] McKibbin W. J. Stoeckel A. 2009 The potential impact of the global financial crisis on world trade. Policy Research Working Paper 5134, World Bank, Washington, DC, November

[RSTB20100131C28] Medvedev D. van der Mensbrugghe D. 2008 Climate change in Latin America: impacts and mitigation policy options. Mimeo, World Bank, Washington, DC, November

[RSTB20100131C29] Orden D. Blandford D. Josling T. 2010 Determinants of farm policies in the United States, 1996–2008. In The political economy of agricultural price distortions, ch. 7 (ed. Anderson K.). Cambridge, UK: Cambridge University Press

[RSTB20100131C30] Pfaffenzeller S. Newbolt P. Rayner A. 2007 A short note on updating the Grilli and Yang commodity price index. World Bank Econ. Rev. 21, 151–163 (doi:10.1093/wber/lhl013)

[RSTB20100131C31] Pfuderer S. Davies G. Mitchell I. 2010 The role of demand for biofuels in the agricultural commodity price spikes of 2007/08. Annex 5 of DEFRA

[RSTB20100131C32] Qaim M. 2009 The economics of genetically modified crops. Annu. Rev. Resour. Econ. 1, 665–693 (doi:10.1146/annurev.resource.050708.144203)

[RSTB20100131C33] Rajagopal D. Sexton S. Hochman G. Zilberman D. 2009 Recent developments in renewable technologies: R&D investment in advanced biofuels. Annu. Rev. Resour. Econ. 1, 621–644 (doi:10.1146/annurev.resource.050708.144259)

[RSTB20100131C34] Reardon T. Timmer C. P. 2007 Transformation of markets for agricultural output in developing countries since 1950: how has thinking changed? In Handbook of agricultural economics, ch. 55, vol. 3 (eds Evenson R. E. Pingali P.). Amsterdam, The Netherlands: Elsevier

[RSTB20100131C35] Reardon T. Barrett C. B. Berdegue J. A. Swinnen J. F. M. 2009 Agrifood industry transformation and farmers in developing countries. World Dev. 37, 1717–1727 (doi:10.1016/j.worlddev.2008.08.023)

[RSTB20100131C36] Rosegrant M. W. Cai X. Cline S. A. 2002 World water and food to 2025. Washington, DC: International Food Policy Research Institute

[RSTB20100131C37] Royal Society 2009 Reaping the benefits: science and the sustainable intensification of global agriculture. London, UK: The Royal Society

[RSTB20100131C38] Sandri D. Valenzuela E. Anderson K. 2007 Economic and trade indicators, 1960 to 2004. Agricultural Distortions Working Paper 02, World Bank, Washington, DC. See www.worldbank.org/agdistortions.www.worldbank.org/agdistortions

[RSTB20100131C39] Smith J. L. 2009 World oil: market or myth? J. Econ. Perspect. 23, 145–164 (doi:10.1257/jep.23.3.145)

[RSTB20100131C40] Swinnen J. F. M. (ed.) 2007 Global supply chains, standards and the poor. London, UK: CABI Publishing

[RSTB20100131C41] Swinnen J. F. M. Vandeplas A. 2009 Market power and rents in global supply chains. In Keynote papers presented in plenary sessions, 27th Conf. of the Int. Association of Agricultural Economists, Beijing, 16–22 August, pp. 149–166

[RSTB20100131C42] Tyers R. Anderson K. 1992 Disarray in world food markets: a quantitative assessment. Cambridge, UK: Cambridge University Press

[RSTB20100131C43] UNCTAD 2009 World investment report 2009: transnational corporations, agricultural production and development. Geneva, Switzerland: United Nations

[RSTB20100131C44] Valenzuela E. van der Mensbrugghe D. Anderson K. 2009 General equilibrium effects of price distortions on global markets, farm incomes and welfare. In Distortions to agricultural incentives: a global perspective, 1955 to 2007 (ed. Anderson K.). London, UK: Palgrave Macmillan; Washington, DC: World Bank

[RSTB20100131C45] von Braun J. Meinzen-Dick R. 2009 ‘Land grabbing’ by foreign investors in developing countries: risks and opportunities. In IFPRI policy brief 13. Washington, DC: International Food Policy Research Institute

[RSTB20100131C46] Wiggins S. Keats S. 2010 Grain stocks and price spikes. Annex 2 of DEFRA

[RSTB20100131C50] World Bank 2008 World development indicators 2008. Washington, DC: World Bank

[RSTB20100131C47] Wright B. D. 2009 International grain reservers and other instruments to address volatility in grain markets. Policy Research Working Paper No. 5028, World Bank, Washington, DC

